# Cancer Stem Cell Metabolism and Potential Therapeutic Targets

**DOI:** 10.3389/fonc.2018.00203

**Published:** 2018-06-05

**Authors:** Vusala Snyder, Tamika C. Reed-Newman, Levi Arnold, Sufi Mary Thomas, Shrikant Anant

**Affiliations:** ^1^Department of Otolaryngology, University of Kansas Medical Center, Kansas City, KS, United States; ^2^Department of General Surgery, University of Kansas Medical Center, Kansas City, KS, United States; ^3^Anatomy and Cell Biology, University of Kansas Medical Center, Kansas City, KS, United States; ^4^Cancer Biology, University of Kansas Medical Center, Kansas City, KS, United States

**Keywords:** stem cells, metabolism, microenvironment, targets, cancer stem cell markers

## Abstract

Malignant tumors contain heterogeneous populations of cells in various states of proliferation and differentiation. The presence of cancer stem or initiating cells is a well-established concept wherein quiescent and poorly differentiated cells within a tumor mass contribute to drug resistance, and under permissive conditions, are responsible for tumor recurrence and metastasis. A number of studies have identified molecular markers that are characteristic of tissue-specific cancer stem cells (CSCs). Isolation of CSCs has enabled studies on the metabolic status of CSCs. As metabolic plasticity is a hallmark of cancer cell adaptation, the intricacies of CSC metabolism and their phenotypic behavior are critical areas of research. Unlike normal stem cells, which rely heavily on oxidative phosphorylation (OXPHOS) as their primary source of energy, or cancer cells, which are primarily glycolytic, CSCs demonstrate a unique metabolic flexibility. CSCs can switch between OXPHOS and glycolysis in the presence of oxygen to maintain homeostasis and, thereby, promote tumor growth. Here, we review key factors that impact CSC metabolic phenotype including heterogeneity of CSCs across different histologic tumor types, tissue-specific variations, tumor microenvironment, and CSC niche. Furthermore, we discuss how targeting key players of glycolytic and mitochondrial pathways has shown promising results in cancer eradication and attenuation of disease recurrence in preclinical models. In addition, we highlight studies on other potential therapeutic targets including complex interactions within the microenvironment and cellular communications in the CSC niche to interfere with CSC growth, resistance, and metastasis.

## Introduction

Despite the advances in modern medicine, some of the major challenges currently confronted in treating cancer patients include the development of therapeutic drug resistance and disease recurrence. Traditional treatments target cancer cells as a means to eradicate tumors and treat the patients. These methods are largely based on the stochastic model—a theory that suggests that cancer cells can arise from a cell that undergoes gene mutations resulting in the acquisition of a highly proliferative state (1, 2). Each progenitor cell bears the mutation and phenotypic profile of the parent cell and is capable of reconstituting a tumor. Several studies have since challenged this theory by demonstrating the existence of a subpopulation of cells called cancer stem cells (CSCs) or tumor-initiating cells, which are typically quiescent but under certain conditions, capable of proliferating to self-renew the CSC population and generate progenitor tumor cells (3, 4). CSCs are resistant to therapies that target rapidly proliferating tumor cells and are primarily responsible for tumor relapse. In the 1990s, the theory of a hierarchical organization within tumors was introduced in acute myeloid leukemia (AML), identifying leukemia-initiating cells *via* their expression of a CD34^++^CD38^−^ phenotype. This hierarchical model postulates that individual tumor cells have distinct mutational profiles and epigenetic modifications contributing to cellular heterogeneity. In the years to follow, researchers have used molecular markers to identify and isolate CSCs of various solid tumors (5–7).

Currently, there are more than 40 established CSC markers (Table 1); however, much controversy surrounds the scientific techniques employed to identify surface markers. Moreover, majority of the markers established for the identification of CSCs were previously described in human embryonic stem cells and/or adult stem cells of normal tissue cells (5, 8). This shared feature may suggest two possibilities: CSCs could originate from genetic alterations in normal stem cells or could be the result of dedifferentiation of mutated cancer cells into stem-like cells. Despite the shared properties, CSCs differ from normal stem cells in that unlike CSCs, cell proliferation is rigidly controlled in normal stem cells (9). Glycosylation of glycoprotein markers has also been suggested to impact the biological behavior of CSCs (8). It is important to focus future investigation on the mutations, metabolic phenotype, and other aspects of the microenvironment that distinguish CSCs from normal stem cells.

**Table 1:** Biomarkers reported to characterize CSCs.

Marker	Cancers identified	Metabolic phenotype	Reference
ABCG2	HNSCC, retinoblastoma, lung cancer, liver cancer, pancreatic cancer, melanoma	Hypoxia induced	(10)

Aldehyde dehydrogenase 1-A1/ALDH1A1	Liver, kidney, red blood cells, skeletal muscle, lung, breast, lens, stomach, brain, pancreas, testis, prostate, ovary	Converts acetaldehyde to acetate, maintains low ROS	(11)

Alpha-methylacyl-CoA racemase/AMACR	Prostate cancer, gastric cancer, nasopharyngeal cancer, CRC	Facilitates metabolic switch to fatty acid β-oxidation	(12)

CD24	Gastric cancer	CD24 is a hypoxia-inducible factor	(13)

CD27	Lymphoma, multiple myeloma, B-cell chronic lymphocytic leukemia, renal cell carcinoma, glioblastoma, mesothelioma, HCC, cancers of the pancreas, breast and ovary, CRC, melanoma, neuro-endocrine carcinoma	Not specified	(14)

CD44	Most epithelial cancers, leukemia	Promotes glycolysis *via* PKM2 suppression	(15)

CD47	AML, ALL, breast cancer, esophageal cancer	Regulates glycolytic metabolic pathways	(16)

CD133	Brain, breast, CRC, HNSCC, kidney, liver, lung, ovary, pancreas, prostate, stomach, bone/soft tissue, eye, skin	Decreased hexokinase II expression, promoted by hypoxia	(17, 18)

Connexin 43/GJA1	Prostate cancer, nasopharyngeal cancer, glioblastoma, HCC	Increased glucose uptake	(19)

c-Met	HNSCC, breast cancer, thyroid cancer, HCC	Prevents excessive ROS	(20, 21)

ErbB2/Her2	Breast cancer, endometrial cancer, gastric cancer	Promotes aerobic glycolysis	(22, 23)

GLI-1	Leukemia, breast cancer, glioma	Hypoxia induced	(24)

GLI-2	Leukemia, breast cancer, glioma, osteosarcoma, HCC, pancreatic cancer	Hypoxia induced	(25)

HIF-2 alpha/EPAS1	HCC, lung cancer, renal cancer, CRC, melanoma, glioblastoma, gastric cancer	Hypoxia induced	(26)

IL-3 R alpha/CD123	AML, pancreatic cancer, non-small cell lung cancer, breast cancer, ovarian cancer	Promotes glycolytic enzyme activity	(27, 28)

IL-6 R alpha	Most epithelial cancers	Promotes glycogenolysis	(29, 30)

Integrin alpha 6/CD49f	Prostate cancer, breast cancer, glioblastoma	Not specified	(31, 32)

Lgr5/GPR49	HNSCC, HCC, CRC, ovarian cancer, basal cell carcinoma	Promotes mitochondrial OXPHOS	(33, 34)

*AML, acute myeloid leukemia; ALL, acute lymphocytic leukemia; CRC, colorectal carcinoma; HNSCC, head and neck squamous cell carcinoma; HCC, hepatocellular carcinoma; OXPHOS, oxidative phosphorylation; ROS, reactive oxygen species; CSCs, cancer stem cells: PKM2, pyruvate kinase M2*.

Normal stem cells are unique in their ability to self-renew, proliferate, and differentiate into various tissue types, as well as reproduce progeny essential to maintain and repair the organ system in which they are found (35, 36). Embryonic stem cells, hematopoietic stem cells, and mesenchymal stem cells have a low mitochondrial DNA copy number, as well as poorly developed mitochondrial morphology and reduced oxidative capacity. On the other hand, glycolytic pathways are highly active in these stem cells. Hypoxia-induced glycolysis in pluripotent stem cells and inhibition of mitochondrial respiration promote stemness, whereas inhibition of glycolysis disrupts proliferation and promotes cell death (37).

Although CSCs share many of the characteristics of normal stem cells, the differ in that, they contribute to tumor progression, drug resistance, and recurrence (38). In addition, several reports suggest that CSCs preferentially use glycolysis. However, other reports suggest a propensity for mitochondrial oxidative phosphorylation (OXPHOS) suggesting a possible metabolic plasticity. The aim of this review is to summarize and emphasize some of the key aspects currently known about CSC metabolism and the potential therapeutic targets contributing to cancer progression.

## Cellular and Cancer Metabolism

All cells require energy for growth, division, and survival, which they acquire through the absorption of nutrients including glucose that are broken down in a series of metabolic reactions involving glycolysis and cellular respiration through OXPHOS. Under normal physiological conditions, cells rely on both glycolysis and OXPHOS for efficient energy production (39). The process of glycolysis involves the breakdown of glucose through a series of reactions to produce pyruvate, two molecules of adenosine 5′-triphosphate (ATP), and nicotinamide adenine dinucleotide (NADH). Under normoxic conditions (oxygen is readily available), pyruvate is transported to the mitochondria where it is converted into acetyl coenzyme A. Acetyl CoA enters the tricarboxylic acid cycle to produce high amounts of energy in the form of NADH and flavin adenine dinucleotide (FADH2) molecules. The hydrogen ions from NADH trigger the electron transport chain and generation of up to 32 molecules of ATP through OXPHOS (40, 41).

Since OXPHOS generates more ATP molecules than glycolysis, normal cells rely primarily on OXPHOS as an efficient source of energy. This process, however, is impaired in hypoxic conditions due a dearth in oxygen.

Rapidly proliferating cancer cells outpace angiogenesis resulting in areas of low oxygen. However, increasing evidence suggests that cancer cells engage in glycolysis even in the presence of oxygen (42). As a result, tumor cells demonstrate enhanced glycolytic production of ATP (43). Otto Warburg first described this phenomenon now known as the Warburg effect of aerobic glycolysis (41, 44, 45). The increased glycolysis was attributed to mitochondrial damage in cancer cells. Subsequent studies found that most cancer cells do not demonstrate mitochondrial damage, but rather suggest that aerobic glycolysis can occur simultaneously to enhance energy production for the maintenance of cancer cell homeostasis (43). In fact, several studies demonstrate that acceleration of glycolysis provides a source of metabolites and other essential factors required for rapidly dividing cells (41, 43, 46, 47).

## Metabolic Phenotype of CSCs

**Figure 1:**
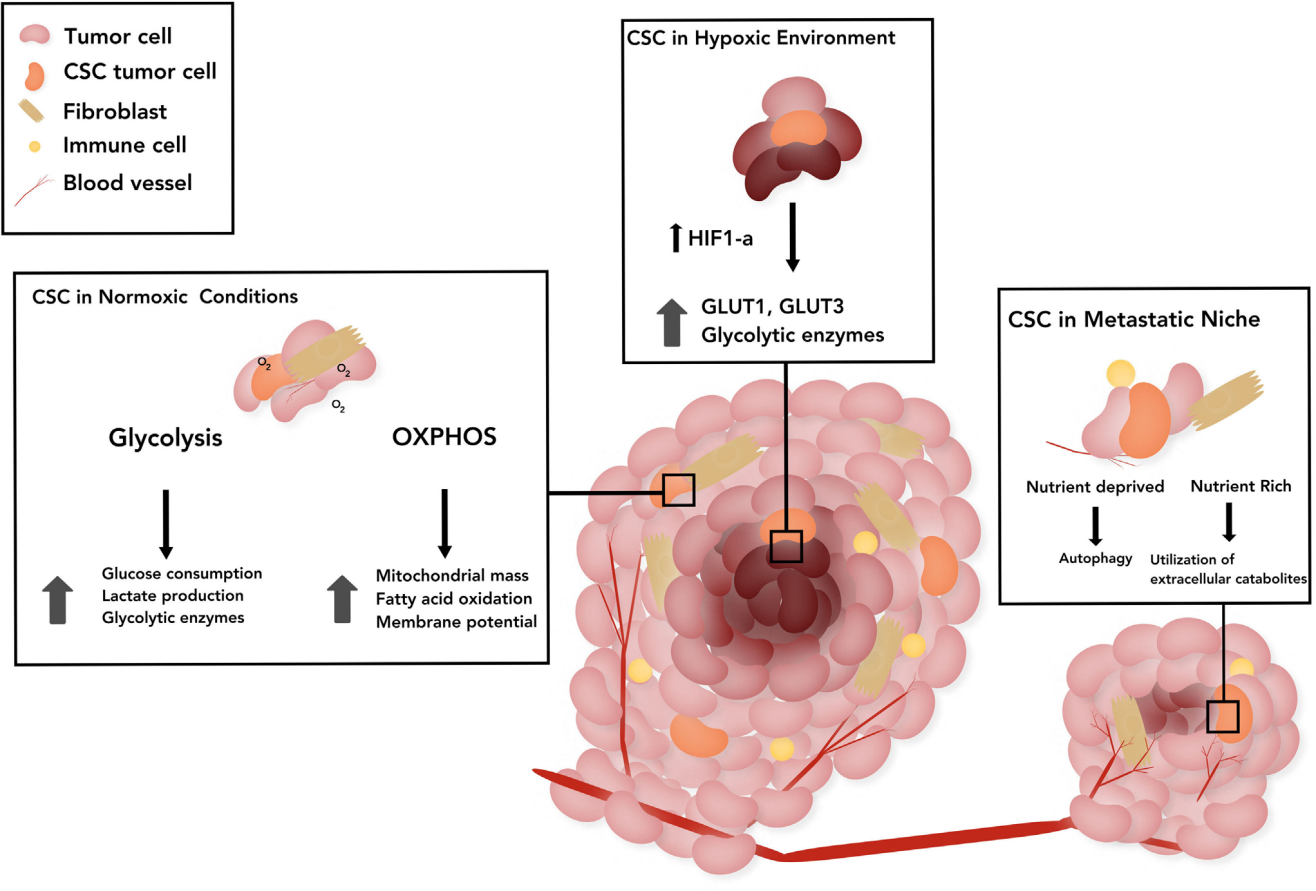
The schematic represents the metabolic status of cancer stem cells (CSCs) in three broad categories based on the location. CSCs in a normoxic tumor: stemness has been associated with upregulation of glycolytic enzymes in those CSCs that rely on glucose pathway, as well as with dependence on mitochondrial pathway as evidenced by increased mitochondrial mass, membrane potential in CSCs and mitochondrial fatty acid oxidation (FAO) for generation of adenosine 5′-triphosphate and nicotinamide adenine dinucleotide. CSCs in tumor under hypoxia: hypoxia-inducible factor-1α (HIF-1α) promotes upregulation and potentiated activity of several glycolytic proteins, such as transporters (GLUT1, GLUT3) and various isoforms of glycolytic enzymes. CSCs in the metastatic niche: CSCs induced by epithelial-to-mesenchymal transition, have augmented utilization of extracellular catabolites, such as pyruvate, lactate, glutamine, glutamate, alanine, or ketone bodies. In nutrient poor states, quiescent disseminated tumor cells rely on alternative energy sources such as autophagy. The CSC stem cell model of treatment proposes the prevention of recurrence if all CSCs are eliminated.

Due to the highly proliferative, tumorigenic, and drug-resistant properties of CSCs, in-depth investigation of CSC metabolic phenotype has comprised the cornerstone of numerous recent studies. Although metabolic adaptation or plasticity is one of the hallmarks of cancer, the majority of reports suggest that CSCs are primarily glycolytic (48–55). However, examination of CSCs isolated from patient tumors suggests that OXPHOS is the main source of energy (56, 57). We describe other multifactorial causes contributing to the apparent differences in CSC metabolism across tumor types in the following sections. Emerging evidence suggests the existence of specific metabolic phenotypes of CSCs based on their location, such as those in actively growing regions of the tumor that have adequate levels of oxygen, hypoxic areas of the tumor, or those in a distant metastatic site summarized in Figure 1.

## Glycolytic Pathway

A number of studies performed in various tumor types, such as glioblastoma, lung cancer, osteosarcoma, breast cancer, ovarian cancer, and colon cancer, suggest that CSCs more strongly favor the glycolytic pathway than other differentiated cancer cells *in vitro* and *in vivo* (58–62). Rationale for investigating the role of glycolytic metabolism in CSCs is due to its proposed phenotypic similarity to normal stem cells with self-renewal characteristics. Earlier studies paved the way by illustrating the low activity of mitochondrial respiration in brain tumor CSCs, as well as higher rates of glycolysis in CSCs than other tumor cells (63, 64). Further investigations revealed that upregulation of glycolytic enzymes (GLUT1, HK-1, and PDK-1) and stimulation of glycolysis are necessary for cell immortalization and is sufficient to increase cellular lifespan (65). Comparing glucose utilization by CSCs and non-CSCs has revealed differentially elevated glucose consumption, lactate synthesis, and ATP content in CSCs, thus suggesting distinct metabolic profiles of CSCs in comparison to non-CSCs (66–68). Glycolysis has also been identified as the preferred metabolic pathway of CSCs in nasopharyngeal carcinoma and of tumor-initiating stem-like cells in hepatocellular carcinoma (69, 70). In addition, cellular metabolism is thought to control stemness characteristics; in particular, the glycolytic switch has a causal relation in induced pluripotent stem cell reprogramming and acquisition of pluripotent markers (71). Reprogramming the metabolic switch from OXPHOS to glycolysis was shown to enhance stemness and CSC properties in CD44^+^CD24^low^EPCAM^+^ cells of basal-like breast cancer by reducing reactive oxygen species (ROS) levels (48). Glycolysis-driven induction of pluripotency is consistent with the finding that hypoxia maintains the stem cell state and a hypoxic environment promotes the reprogramming process (72).

## Oxphos Pathway

Growing evidence suggests mitochondrial oxidative metabolism as the preferred form of energy production in CSCs. Several studies in numerous tumor types, such as CD133^+^ cells of glioblastoma and pancreatic ductal adenocarcinoma, ROS^low^ quiescent leukemia stem cells, lung cancer side population cells, and breast cancer, strongly support an OXPHOS phenotype and less glycolytic profile (49, 50, 54, 73). In contrast to the non-CSC cancer cells, which mainly utilize glycolysis for energy production, CSCs have an enhanced mitochondrial ROS, higher rates of oxygen consumption, and overall increased mitochondrial function, as evidenced by increased mitochondrial mass and membrane potential (50, 52, 53, 73–76). Moreover, this increased mitochondrial bulk in a subpopulation of breast cancer cells induces stem-like characteristics and confers metastatic potential and resistance to DNA damage (77). In addition, CSCs may depend on mitochondrial fatty acid oxidation (FAO) for the generation of ATP and NADH. A population of isolated ovarian CSCs revealed upregulated expression of genes associated with FAO and OXPHOS (52). FAO is instrumental in self-renewal processes of hematopoietic stem cells and leukemia-initiating cells, as in the survival of ablation-resistant pancreatic CSCs and survival of epithelial cancer cells subsequent to matrix detachment (78–80). An oxidative phenotype confers resistance to treatment modalities and evasion of apoptosis as evidenced by the vastly tumorigenic and chemoresistant metabolism found in hepatocellular CSCs, upon NANOG-induced expression of FAO genes (70). The powerful antioxidant defense mechanism of CSCs contributes to therapy resistance, by maintaining a significantly lower ROS levels and preserving stemness and tumorigenic properties of CSCs (52, 81, 82).

## Factors Affecting the Metabolic Status of CSCs

The reported differences in the metabolic profile of CSCs from various tumor types are due to multifactorial causes. One such explanation is the suggested plasticity of these cells and the potential harvest of them at various stages of differentiation/dedifferentiation during experiments (2). Another cause may be the lack of uniformity and precision in definition of CSCs and varying techniques utilized to isolate CSCs, such as specific markers, Hoechst staining-based sorting, chemoresistance-based isolation, and reoxygenation sorting post-hypoxic exposure (83–87). This is due to a vast heterogeneity of CSCs across various histologic tumor types. Another potential contributing factor that may explain the contradictory results is the contribution of the microenvironment. We broadly distinguish the metabolic status of CSCs in three locations, namely, regions with normoxic tumor, hypoxic tumor, and at metastatic sites (Figure 1).

As mentioned in the preceding sections, under normoxic conditions, CSCs can engage in glycolysis and/or OXPHOS. Furthermore, the metabolic status of CSCs can be affected by crosstalk between CSCs and cancer-associated stroma in the microenvironment. For example, cancer-associated fibroblast-secreted metabolites including lactate and ketone bodies drive OXPHOS in cancer cells (88). The role of cancer-associated stroma in regulating CSC metabolism is unknown.

Similar to embryonic stem cell maintenance, tumor hypoxia promotes the persistence of an undifferentiated, stem cell phenotype (89). Ductal breast carcinoma cells revert to a stem cell-like phenotype through dedifferentiation under hypoxic conditions (90). Other studies showed that hypoxic exposure altered gene expression in human neuroblastoma cells toward a neural crest-like, immature profile and caused upregulation of the stem cell surface marker CD-133 in medulloblastoma (90, 91). Under hypoxic conditions, overexpression of hypoxia-inducible factor-1α (HIF-1α) promotes upregulation and potentiated activity of several glycolytic proteins, such as transporters (GLUT1 and GLUT3) and various isoforms of glycolytic enzymes (92). In addition, HIF-1α regulates pyruvate dehydrogenase kinase 1 levels which facilitate glycolysis in breast CSCs under hypoxic conditions (93).

Metastatic cancer cells undergo epithelial-to-mesenchymal transition (EMT) upregulating a number of factors associated with a stem-like phenotype. EMT-associated factors including HIF-1α, Wnt, and Snail regulate cellular metabolism (94). Furthermore, EMT-associated metabolites—glutamine, glutamate, and alanine—as well as high lactate concentrations are associated with poor survival and higher metastatic potential in breast cancers (95, 96). CSCs have augmented utilization of extracellular catabolites, such as pyruvate, lactate, glutamine, glutamate, alanine, or ketone bodies to support OXPHOS (97–99). In nutrient poor states, quiescent disseminated tumor cells rely on alternative energy sources such as autophagy, yet metabolic plasticity demonstrated by their ability to produce energy through various pathways is instrumental for metastatic growth and proliferation (100–102). Finally, a recent study of metabolic dependencies of non-small cell lung cancers highlighted the significant contribution of the microenvironment as a determinant of the metabolic phenotype of cancer cells, as evidenced by varying profiles *in vitro* and *in vivo* settings. *KRAS*-driven lung cancer cells in mice models showed preferential glutamine utilization *in vitro*, but did not depend on glutamine metabolism *in vivo* (103).

## Targeting Cellular Metabolism

A strong association between tumors with high CSC fractions and recurrence, poorer overall survival, and higher incidence of metastasis, underscores the significant prognostic and therapeutic implications of CSCs (104, 105). Defining characteristics of CSCs such as surface markers, metabolic phenotypes, resistance to chemoradiotherapy, and regulatory factors in microenvironment compile the bulk of therapeutic targets. For instance, CD44, a receptor for hyaluronic acid-mediated motility, is shown to induce CSC attachment to extracellular matrix and cell migration, promoting metastasis and invasion (106). Treatment of breast, colon, esophageal, gastric, lung, and ovarian cancers overexpressing CD44, with ONCOFID^TM^-S which is a conjugate of hyaluron and chemotherapeutic agent SN38 (7-ethyl-10-hydroxycamptothecin, active metabolite of CPT-11) revealed a strong *in vitro* anti-proliferative activity (107, 108). In addition, use of anti-CD44 antibodies H90 and A3D8 inhibited proliferation and induced apoptosis, by promoting the differentiation of AML blasts (108–111). Finally, CD44 interacts with pyruvate kinase M2 (PKM2), enhancing the glycolytic profile of cancer cells deficient in p53 or exposed to hypoxia. Subsequent ablation of CD44 led to inhibition of glycolysis, increase in ROS and enhancement of chemotherapeutic drug effect in these cancer cells (110). Therefore, preferentially targeting of identified CSC markers, such as CD44, can be utilized for an effective cytotoxic drug delivery. In addition, inhibition of glycolysis can be achieved by targeting various glycolytic enzymes, transporters, and other complex regulators, such as GLUT 1–4, hexokinase, PKM2, and lactate dehydrogenase A (111–113).

Previously discussed evidence for OXPHOS dependence of CSCs in numerous cancer lines proposes mitochondrial metabolism to be a potential target for an effective elimination of CSCs. Inhibition of the OXPHOS pathway reduces sphere formation and tumor formation potential demonstrating vulnerability of CSC to mitochondria-targeted therapies (54, 114, 115). Pharmacological agents targeting CSCs through inhibition of mitochondrial biogenesis and OXPHOS are currently under investigation for cancer treatment. Several FDA-approved compounds known to inhibit mitochondrial function have been reported to achieve a more effective eradication of CSCs. Salinomycin, erythromycins, tetracyclines, and glycylcyclines are some of the approved agents to have already demonstrated efficacy in eradicating CSCs *via* reduction of stemness properties (115–118). Metformin, an inhibitor of OXPHOS complex I, has demonstrated anti-tumoral activity by reducing mammosphere formation, delaying *in vivo* tumor growth, and inducing apoptosis in pancreatic CSCs unable to switch to glycolysis (54, 119, 120). However, emergence of a small subset of resistant CSCs with an intermediate glycolytic/OXPHOS phenotype could be prevented/reversed by utilizing a mitochondrial ROS inducer such as menadione (54). Dual mechanism of menadione inhibition of Complex I and induction of mitochondrial ROS points out the superior efficacy of multi-modal targeted therapy. Studies have shown that inhibition of mitochondrial respiration not only induces apoptosis in pancreatic CSCs with OXPHOS phenotype but also effectively eliminates primarily glycolytic breast and nasopharyngeal CSCs (53, 54, 121). These data highlight the extended role of mitochondria beyond energy production in CSCs, such as acquiring metabolites from glutamine *via* reductive carboxylation to support growth in tumor cells with defective mitochondria (122). A novel compound 3,5-bis(2,4-difluorobenzylidene)-4-piperidone (DiFiD) has been shown to inhibit pancreatic cancer growth by targeting a CSC marker, doublecortin and CaM kinase-like-1 (DCLK-1) (59, 66, 123). However, the role of DCLK-1 and the impact of DiFiD on CSC metabolism have not been studied.

Evident from the data reviewed, the CSC phenotype varies between cancer subtypes and among populations of the same subtypes. Preferred energy-producing metabolic pathways depend on various factors, including metastatic site highlighting vast metabolic variability and patterns (124). In addition to studies supporting metabolic plasticity, simultaneous enhancement of glycolysis and OXPHOS pathways was observed in highly metastatic breast cancer cell lines relative to non-metastatic cell lines (49, 124). Consequently, dual inhibition of glycolytic and mitochondrial energy pathways has proven to be effective against tumor growth in a number of preclinical cancer models (125). One such study elegantly demonstrated sarcoma cells to be twofold to fivefold more sensitive than normal cells to dual inhibition of glycolysis with 2-deoxyglucose and OXPHOS with oligomycin or metformin (126). Therefore, dual inhibition of metabolic pathways may be a superior approach to eradicating heterogeneous CSCs rather than singularly targeting glycolysis or OXPHOS pathways. Finally, other factors directly affecting metabolic status of CSCs may represent potential targets for pharmacological treatments. These developments may include promoting CSC differentiation, targeting complex interactions within the microenvironment, and disrupting cellular communications in the CSC niche to interfere with CSC growth, resistance, and metastasis (97, 127–130).

## Conclusion

Substantial evidence suggests that the CSCs are pluripotent, self-renewing, “original cells” of a tumor capable of differentiation into more specialized cancer cell types. CSCs are responsible for tumor formation, differentiation, maintenance, spread, and recurrence, making them an attractive therapeutic target for a potential permanent cure or long-term disease-free survival (127, 131). Regardless of the controversy about the metabolic phenotype of CSCs, metabolism is not only a key player but also a regulatory instigator of stemness.

Metabolic singularities that distinguish CSCs need to be further investigated, as they offer a great potential for developing improved treatments to eradicate them. In particular, streamlining and standardization of CSC identification methods is important. Development of CSC marker combinations would contribute to better delineation of CSCs from non-CSC cancer cells and normal stem cells. Interactions between CSCs and their microenvironment also provide a fertile ground for advance investigations. Chronicity and causality of these complex interactions needs to be established. Moving forward, CSC metabolic pathways and principal players of metabolism comprise potential therapeutic targets with a great promise for improved cancer treatments.

## Author Contributions

VS carried out the literature review and wrote and edited the manuscript. TR-N carried out the literature review and wrote a section of the manuscript. LA carried out literature review and prepared the figure and table included in the manuscript. SA and ST conceptualized the manuscript, supervised the writing, and edited the manuscript.

## Conflict of Interest Statement

The authors declare that the research was conducted in the absence of any commercial or financial relationships that could be construed as a potential conflict of interest.

## References

[1] Odoux C Fohrer H Hoppo T Guzik L Stolz DB Lewis DW A stochastic model for cancer stem cell origin in metastatic colon cancer. Cancer Res (2008) 68(17):6932–41. 10.1158/0008-5472.CAN-07-5779 18757407 PMC2562348

[2] Peiris-Pagès M Martinez-Outschoorn UE Pestell RG Sotgia F Lisanti MP. Cancer stem cell metabolism. Breast Cancer Res (2016) 18(1):55. 10.1186/s13058-016-0712-6 27220421 PMC4879746

[3] Badri H Leder K. Optimal treatment and stochastic modeling of heterogeneous tumors. Biol Direct (2016) 11:40. 10.1186/s13062-016-0142-5 27549860 PMC4994177

[4] Kreso A Dick JE. Evolution of the cancer stem cell model. Cell Stem Cell (2014) 14(3):275–91. 10.1016/j.stem.2014.02.006 24607403

[5] Kim WT Ryu CJ. Cancer stem cell surface markers on normal stem cells. BMB Rep (2017) 50(6):285–98. 10.5483/BMBRep.2017.50.6.039 28270302 PMC5498139

[6] Zheng S Xin L Liang A Fu Y. Cancer stem cell hypothesis: a brief summary and two proposals. Cytotechnology (2013) 65(4):505–12. 10.1007/s10616-012-9517-3 23250634 PMC3720968

[7] Langenkamp U Siegler U Jörger S Diermayr S Gratwohl A Kalberer CP Human acute myeloid leukemia CD34(+)CD38(−) stem cells are susceptible to allorecognition and lysis by single KIR-expressing natural killer cells. Haematologica (2009) 94(11):1590–4. 10.3324/haematol.2009.005967 19608675 PMC2770970

[8] Karsten U Goletz S. What makes cancer stem cell markers different? Springerplus (2013) 2(1):301. 10.1186/2193-1801-2-301 23888272 PMC3710573

[9] Li L Neaves WB. Normal stem cells and cancer stem cells: the niche matters. Cancer Res (2006) 66(9):4553–7. 10.1158/0008-5472.CAN-05-3986 16651403

[10] Ding XW Wu JH Jiang CP. ABCG2: a potential marker of stem cells and novel target in stem cell and cancer therapy. Life Sci (2010) 86(17):631–7. 10.1016/j.lfs.2010.02.012 20159023

[11] Tomita H Tanaka K Tanaka T Hara A. Aldehyde dehydrogenase 1A1 in stem cells and cancer. Oncotarget (2016) 7(10):11018–32. 10.18632/oncotarget.6920 26783961 PMC4905455

[12] Lloyd MD Yevglevskis M Lee GL Wood PJ Threadgill MD Woodman TJ. α-Methylacyl-CoA racemase (AMACR): metabolic enzyme, drug metabolizer and cancer marker P504S. Progr Lipid Res (2013) 52(2):220–30. 10.1016/j.plipres.2013.01.001 23376124

[13] Fujikuni N Yamamoto H Tanabe K Naito Y Sakamoto N Tanaka Y Hypoxia-mediated CD24 expression is correlated with gastric cancer aggressiveness by promoting cell migration and invasion. Cancer Sci (2014) 105(11):1411–20. 10.1111/cas.12522 25174257 PMC4462374

[14] Jacobs J Deschoolmeester V Zwaenepoel K Rolfo C Silence K Rottey S CD70: an emerging target in cancer immunotherapy. Pharmacol Ther (2015) 155:1–10. 10.1016/j.pharmthera.2015.07.007 26213107

[15] Yan Y Zuo X Wei D. Concise review: emerging role of CD44 in cancer stem cells: a promising biomarker and therapeutic target. Stem Cells Transl Med (2015) 4(9):1033–43. 10.5966/sctm.2015-0048 26136504 PMC4542874

[16] Suzuki S Yokobori T Tanaka N Sakai M Sano A Inose T CD47 expression regulated by the miR-133a tumor suppressor is a novel prognostic marker in esophageal squamous cell carcinoma. Oncol Rep (2012) 28(2):465–72. 10.3892/or.2012.1831 22641236

[17] Grosse-Gehling P Fargeas CA Dittfeld C Garbe Y Alison MR Corbeil D CD133 as a biomarker for putative cancer stem cells in solid tumours: limitations, problems and challenges. J Pathol (2013) 229(3):355–78. 10.1002/path.4086 22899341

[18] Li Z. CD133: a stem cell biomarker and beyond. Exp Hematol Oncol (2013) 2:17. 10.1186/2162-3619-2-17 23815814 PMC3701589

[19] Wang W-K Chen M-C Leong H-F Kuo Y-L Kuo C-Y Lee C-H Connexin 43 suppresses tumor angiogenesis by down-regulation of vascular endothelial growth factor via hypoxic-induced factor-1α. Int J Mol Sci (2015) 16(1):439–51. 10.3390/ijms16010439 PMC4307255 25548899

[20] Lim YC Kang HJ Moon JH. C-Met pathway promotes self-renewal and tumorigenecity of head and neck squamous cell carcinoma stem-like cell. Oral Oncol (2014) 50(7):633–9. 10.1016/j.oraloncology.2014.04.004 24835851

[21] Gómez-Quiroz LE Factor VM Kaposi-Novak P Coulouarn C Conner EA Thorgeirsson SS. Hepatocyte-specific c-Met deletion disrupts redox homeostasis and sensitizes to Fas-mediated apoptosis. J Biol Chem (2008) 283(21):14581–9. 10.1074/jbc.M707733200 18348981 PMC2386934

[22] Contino F Mazzarella C Ferro A Lo Presti M Roz E Lupo C Negative transcriptional control of ERBB2 gene by MBP-1 and HDAC1: diagnostic implications in breast cancer. BMC Cancer (2013) 13:81. 10.1186/1471-2407-13-81 23421821 PMC3599235

[23] Walsh A Cook RS Rexer B Arteaga CL Skala MC. Optical imaging of metabolism in HER2 overexpressing breast cancer cells. Biomed Opt Express (2012) 3(1):75–85. 10.1364/BOE.3.000075 22254170 PMC3255344

[24] Lei J Fan L Wei G Chen X Duan W Xu Q Gli-1 is crucial for hypoxia-induced epithelial-mesenchymal transition and invasion of breast cancer. Tumor Biol (2015) 36(4):3119–26. 10.1007/s13277-014-2948-z 25501705

[25] Im S Choi HJ Yoo C Jung JH Jeon YW Suh YJ Hedgehog related protein expression in breast cancer: Gli-2 is associated with poor overall survival. Korean J Pathol (2013) 47(2):116–23. 10.4132/KoreanJPathol.2013.47.2.116 23667370 PMC3647123

[26] Zhao J Du F Luo Y Shen G Zheng F Xu B. The emerging role of hypoxia-inducible factor-2 involved in chemo/radioresistance in solid tumors. Cancer Treat Rev (2015) 41(7):623–33. 10.1016/j.ctrv.2015.05.004 25981453

[27] Dentelli P Rosso A Olgasi C Camussi G Brizzi MF. IL-3 is a novel target to interfere with tumor vasculature. Oncogene (2011) 30(50):4930–40. 10.1038/onc.2011.204 21643009

[28] Bauer DE Harris MH Plas DR Lum JJ Hammerman PS Rathmell JC Cytokine stimulation of aerobic glycolysis in hematopoietic cells exceeds proliferative demand. FASEB J (2004) 18(11):1303–5. 10.1096/fj.03-1001fje 15180958 PMC4458073

[29] Krishnamurthy S Warner KA Dong Z Imai A Nör C Ward BB Endothelial interleukin-6 defines the tumorigenic potential of primary human cancer stem cells. Stem Cells (2014) 32(11):2845–57. 10.1002/stem.1793 25078284 PMC4198458

[30] Mauer J Denson JL Brüning JC. Versatile functions for IL-6 in metabolism and cancer. Trends Immunol (2015) 36(2):92–101. 10.1016/j.it.2014.12.008 25616716

[31] Kacsinta AD Rubenstein CS Sroka IC Pawar S Gard JM Nagle RB Intracellular modifiers of integrin alpha 6p production in aggressive prostate and breast cancer cell lines. Biochem Biophys Res Commun (2014) 454(2):335–40. 10.1016/j.bbrc.2014.10.073 25450398 PMC4254650

[32] Lathia JD Gallagher J Heddleston JM Wang J Eyler CE Macswords J Integrin alpha 6 regulates glioblastoma stem cells. Cell Stem Cell (2010) 6(5):421–32. 10.1016/j.stem.2010.02.018 20452317 PMC2884275

[33] Chen Q Zhang X Li WM Ji YQ Cao HZ Zheng P. Prognostic value of LGR5 in colorectal cancer: a meta-analysis. PLoS One (2014) 9(9):e107013. 10.1371/journal.pone.0107013 25192390 PMC4156381

[34] Rodríguez-Colman MJ Schewe M Meerlo M Stigter E Gerrits J Pras-Raves M Interplay between metabolic identities in the intestinal crypt supports stem cell function. Nature (2017) 543(7645):424–7. 10.1038/nature21673 28273069

[35] Botelho M Alves H. Significance of cancer stem cells in anti-cancer therapies. Int J Immunother Cancer Res (2016) 2(1):14–6. 10.17352/2455-8591.000010 28191547 PMC5298826

[36] NIH Stem Cell Information Home Page. Stem Cell Information. Bethesda, MD: National Institutes of Health, U.S. Department of Health and Human Services (2016). Available from: https://stemcells.nih.gov/info/basics/1.htm (Accessed: May 28, 2018).

[37] Folmes CD Dzeja PP Nelson TJ Terzic A. Metabolic plasticity in stem cell homeostasis and differentiation. Cell Stem Cell (2012) 11(5):596–606. 10.1016/j.stem.2012.10.002 23122287 PMC3593051

[38] Rahman M Deleyrolle L Vedam-Mai V Azari H Abd-El-Barr M Reynolds BA. The cancer stem cell hypothesis: failures and pitfalls. Neurosurgery (2011) 68(2):531–45; discussion 545. 10.1227/NEU.0b013e3181ff9eb5 21135745

[39] Baudot A de la Torre V Valencia A. Mutated genes, pathways and processes in tumours. EMBO Rep (2010) 11(10):805–10. 10.1038/embor.2010.133 20847737 PMC2948187

[40] Sancho P Barneda D Heeschen C. Hallmarks of cancer stem cell metabolism. Br J Cancer (2016) 114(12):1305–12. 10.1038/bjc.2016.152 27219018 PMC4984474

[41] San-Millan I Brooks GA. Reexamining cancer metabolism: lactate production for carcinogenesis could be the purpose and explanation of the Warburg effect. Carcinogenesis (2017) 38(2):119–33. 10.1093/carcin/bgw127 27993896 PMC5862360

[42] Reya T Morrison SJ Clarke MF Weissman IL. Stem cells, cancer, and cancer stem cells. Nature (2001) 414(6859):105–11. 10.1038/35102167 11689955

[43] Vander Heiden MG Cantley LC Thompson CB. Understanding the Warburg effect: the metabolic requirements of cell proliferation. Science (2009) 324(5930):1029–33. 10.1126/science.1160809 19460998 PMC2849637

[44] Warburg O On the origin of cancer cells. Science (1956) 123(3191):309–14. 10.1126/science.123.3191.309 13298683

[45] Jang M Kim SS Lee J. Cancer cell metabolism: implications for therapeutic targets. Exp Mol Med (2013) 45(10):e45. 10.1038/emm.2013.85 24091747 PMC3809361

[46] Lee M Yoon JH. Metabolic interplay between glycolysis and mitochondrial oxidation: the reverse Warburg effect and its therapeutic implication. World J Biol Chem (2015) 6(3):148–61. 10.4331/wjbc.v6.i3.148 26322173 PMC4549759

[47] De Preter G Neveu MA Danhier P Brisson L Payen VL Porporato PE Inhibition of the pentose phosphate pathway by dichloroacetate unravels a missing link between aerobic glycolysis and cancer cell proliferation. Oncotarget (2016) 7(3):2910–20. 10.18632/oncotarget.6272 26543237 PMC4823080

[48] Dong C Yuan T Wu Y Wang Y Fan TW Miriyala S Loss of FBP1 by Snail-mediated repression provides metabolic advantages in basal-like breast cancer. Cancer Cell (2013) 23(3):316–31. 10.1016/j.ccr.2013.01.022 23453623 PMC3703516

[49] Ye XQ Li Q Wang GH Sun FF Huang GJ Bian XW Mitochondrial and energy metabolism-related properties as novel indicators of lung cancer stem cells. Int J Cancer (2011) 129(4):820–31. 10.1002/ijc.25944 21520032

[50] Janiszewska M Suvà ML Riggi N Houtkooper RH Auwerx J Clément-Schatlo V Imp2 controls oxidative phosphorylation and is crucial for preserving glioblastoma cancer stem cells. Genes Dev (2012) 26(17):1926–44. 10.1101/gad.188292.112 22899010 PMC3435496

[51] Anderson AS Roberts PC Frisard MI Hulver MW Schmelz EM. Ovarian tumor-initiating cells display a flexible metabolism. Exp Cell Res (2014) 328(1):44–57. 10.1016/j.yexcr.2014.08.028 25172556 PMC4260041

[52] Pasto A Bellio C Pilotto G Ciminale V Silic-Benussi M Guzzo G Cancer stem cells from epithelial ovarian cancer patients privilege oxidative phosphorylation, and resist glucose deprivation. Oncotarget (2014) 5(12):4305–19. 10.18632/oncotarget.2010 24946808 PMC4147325

[53] Shen YA Lin CH Chi WH Wang CY Hsieh YT Wei YH Resveratrol impedes the stemness, epithelial-mesenchymal transition, and metabolic reprogramming of cancer stem cells in nasopharyngeal carcinoma through p53 activation. Evid Based Complement Alternat Med (2013) 2013:590393. 10.1155/2013/590393 23737838 PMC3657420

[54] Sancho P Burgos-Ramos E Tavera A Bou Kheir T Jagust P Schoenhals M MYC/PGC-1alpha balance determines the metabolic phenotype and plasticity of pancreatic cancer stem cells. Cell Metab (2015) 22(4):590–605. 10.1016/j.cmet.2015.08.015 26365176

[55] Dando I Dalla Pozza E Biondani G Cordani M Palmieri M Donadelli M. The metabolic landscape of cancer stem cells. IUBMB Life (2015) 67(9):687–93. 10.1002/iub.1426 26337609

[56] Feng W Gentles A Nair RV Huang M Lin Y Lee CY Targeting unique metabolic properties of breast tumor initiating cells. Stem Cells (2014) 32(7):1734–45. 10.1002/stem.1662 24497069 PMC4144791

[57] Vlashi E Lagadec C Vergnes L Matsutani T Masui K Poulou M Metabolic state of glioma stem cells and nontumorigenic cells. Proc Natl Acad Sci U S A (2011) 108(38):16062–7. 10.1073/pnas.1106704108 21900605 PMC3179043

[58] Ciavardelli D Rossi C Barcaroli D Volpe S Consalvo A Zucchelli M Breast cancer stem cells rely on fermentative glycolysis and are sensitive to 2-deoxyglucose treatment. Cell Death Dis (2014) 5:e1336. 10.1038/cddis.2014.285 25032859 PMC4123079

[59] Liao J Qian F Tchabo N Mhawech-Fauceglia P Beck A Qian Z Ovarian cancer spheroid cells with stem cell-like properties contribute to tumor generation, metastasis and chemotherapy resistance through hypoxia-resistant metabolism. PLoS One (2014) 9(1):e84941. 10.1371/journal.pone.0084941 24409314 PMC3883678

[60] Palorini R Votta G Balestrieri C Monestiroli A Olivieri S Vento R Energy metabolism characterization of a novel cancer stem cell-like line 3AB-OS. J Cell Biochem (2014) 115(2):368–79. 10.1002/jcb.24671 24030970

[61] Zhou Y Zhou Y Shingu T Feng L Chen Z Ogasawara M Metabolic alterations in highly tumorigenic glioblastoma cells: preference for hypoxia and high dependency on glycolysis. J Biol Chem (2011) 286(37):32843–53. 10.1074/jbc.M111.260935 21795717 PMC3173179

[62] Chen KY Liu X Bu P Lin CS Rakhilin N Locasale JW A metabolic signature of colon cancer initiating cells. Conf Proc IEEE Eng Med Biol Soc (2014) 2014:4759–62. 10.1109/EMBC.2014.6944688 25571056 PMC4302416

[63] Wu M Neilson A Swift AL Moran R Tamagnine J Parslow D Multiparameter metabolic analysis reveals a close link between attenuated mitochondrial bioenergetic function and enhanced glycolysis dependency in human tumor cells. Am J Physiol Cell Physiol (2007) 292(1):C125–36. 10.1152/ajpcell.00247.2006 16971499

[64] Liu PP Liao J Tang ZJ Wu WJ Yang J Zeng ZL Metabolic regulation of cancer cell side population by glucose through activation of the Akt pathway. Cell Death Differ (2014) 21(1):124–35. 10.1038/cdd.2013.131 24096870 PMC3857620

[65] Kondoh H Lleonart ME Gil J Wang J Degan P Peters G Glycolytic enzymes can modulate cellular life span. Cancer Res (2005) 65(1):177–85. 15665293

[66] Emmink BL Verheem A Van Houdt WJ Steller EJ Govaert KM Pham TV The secretome of colon cancer stem cells contains drug-metabolizing enzymes. J Proteomics (2013) 91:84–96. 10.1016/j.jprot.2013.06.027 23835434

[67] Hammoudi N Ahmed KB Garcia-Prieto C Huang P. Metabolic alterations in cancer cells and therapeutic implications. Chin J Cancer (2011) 30(8):508–25. 10.5732/cjc.011.10267 21801600 PMC4013402

[68] Liu Y Meyer C Xu C Weng H Hellerbrand C ten Dijke P Animal models of chronic liver diseases. Am J Physiol Gastrointest Liver Physiol (2013) 304(5):G449–68. 10.1152/ajpgi.00199.2012 23275613

[69] Shen YA Wang CY Hsieh YT Chen YJ Wei YH. Metabolic reprogramming orchestrates cancer stem cell properties in nasopharyngeal carcinoma. Cell Cycle (2015) 14(1):86–98. 10.4161/15384101.2014.974419 25483072 PMC4352969

[70] Chen CL Uthaya Kumar DB Punj V Xu J Sher L Tahara SM NANOG metabolically reprograms tumor-initiating stem-like cells through tumorigenic changes in oxidative phosphorylation and fatty acid metabolism. Cell Metab (2016) 23(1):206–19. 10.1016/j.cmet.2015.12.004 26724859 PMC4715587

[71] Folmes CD Nelson TJ Martinez-Fernandez A Arrell DK Lindor JZ Dzeja PP Somatic oxidative bioenergetics transitions into pluripotency-dependent glycolysis to facilitate nuclear reprogramming. Cell Metab (2011) 14(2):264–71. 10.1016/j.cmet.2011.06.011 21803296 PMC3156138

[72] Mohyeldin A Garzon-Muvdi T Quinones-Hinojosa A. Oxygen in stem cell biology: a critical component of the stem cell niche. Cell Stem Cell (2010) 7(2):150–61. 10.1016/j.stem.2010.07.007 20682444

[73] Lagadinou ED Sach A Callahan K Rossi RM Neering SJ Minhajuddin M BCL-2 inhibition targets oxidative phosphorylation and selectively eradicates quiescent human leukemia stem cells. Cell Stem Cell (2013) 12(3):329–41. 10.1016/j.stem.2012.12.013 23333149 PMC3595363

[74] De Luca A Fiorillo M Peiris-Pagès M Ozsvari B Smith DL Sanchez-Alvarez R Mitochondrial biogenesis is required for the anchorage-independent survival and propagation of stem-like cancer cells. Oncotarget (2015) 6(17):14777–95. 10.18632/oncotarget.4401 26087310 PMC4558115

[75] Lamb R Bonuccelli G Ozsvári B Peiris-Pagès M Fiorillo M Smith DL Mitochondrial mass, a new metabolic biomarker for stem-like cancer cells: understanding WNT/FGF-driven anabolic signaling. Oncotarget (2015) 6(31):30453–71. 10.18632/oncotarget.5852 26421711 PMC4741544

[76] Vlashi E Lagadec C Vergnes L Reue K Frohnen P Chan M Metabolic differences in breast cancer stem cells and differentiated progeny. Breast Cancer Res Treat (2014) 146(3):525–34. 10.1007/s10549-014-3051-2 25007966 PMC4131557

[77] Farnie G Sotgia F Lisanti MP. High mitochondrial mass identifies a sub-population of stem-like cancer cells that are chemo-resistant. Oncotarget (2015) 6(31):30472–86. 10.18632/oncotarget.5401 26421710 PMC4741545

[78] Samudio I Harmancey R Fiegl M Kantarjian H Konopleva M Korchin B Pharmacologic inhibition of fatty acid oxidation sensitizes human leukemia cells to apoptosis induction. J Clin Invest (2010) 120(1):142–56. 10.1172/JCI38942 20038799 PMC2799198

[79] Ito K Carracedo A Weiss D Arai F Ala U Avigan DE A PML-PPAR-delta pathway for fatty acid oxidation regulates hematopoietic stem cell maintenance. Nat Med (2012) 18(9):1350–8. 10.1038/nm.2882 22902876 PMC3566224

[80] Prigione A Fauler B Lurz R Lehrach H Adjaye J. The senescence-related mitochondrial/oxidative stress pathway is repressed in human induced pluripotent stem cells. Stem Cells (2010) 28(4):721–33. 10.1002/stem.404 20201066

[81] Schafer ZT Grassian AR Song L Jiang Z Gerhart-Hines Z Irie HY Antioxidant and oncogene rescue of metabolic defects caused by loss of matrix attachment. Nature (2009) 461(7260):109–13. 10.1038/nature08268 19693011 PMC2931797

[82] Diehn M Cho RW Lobo NA Kalisky T Dorie MJ Kulp AN Association of reactive oxygen species levels and radioresistance in cancer stem cells. Nature (2009) 458(7239):780–3. 10.1038/nature07733 19194462 PMC2778612

[83] Tang C Ang BT Pervaiz S. Cancer stem cell: target for anti-cancer therapy. FASEB J (2007) 21(14):3777–85. 10.1096/fj.07-8560rev 17625071

[84] Bomken S Fiser K Heidenreich O Vormoor J. Understanding the cancer stem cell. Br J Cancer (2010) 103(4):439–45. 10.1038/sj.bjc.6605821 20664590 PMC2939794

[85] Schatton T Frank NY Frank MH. Identification and targeting of cancer stem cells. Bioessays (2009) 31(10):1038–49. 10.1002/bies.200900058 19708024 PMC2887758

[86] Li HZ Yi TB Wu ZY. Suspension culture combined with chemotherapeutic agents for sorting of breast cancer stem cells. BMC Cancer (2008) 8:135. 10.1186/1471-2407-8-135 18477410 PMC2390573

[87] Louie E Nik S Chen JS Schmidt M Song B Pacson C Identification of a stem-like cell population by exposing metastatic breast cancer cell lines to repetitive cycles of hypoxia and reoxygenation. Breast Cancer Res (2010) 12(6):R94. 10.1186/bcr2773 21067584 PMC3046435

[88] Zhang D Wang Y Shi Z Liu J Sun P Hou X Metabolic reprogramming of cancer-associated fibroblasts by IDH3alpha downregulation. Cell Rep (2015) 10(8):1335–48. 10.1016/j.celrep.2015.02.006 25732824

[89] Quail DF Taylor MJ Postovit LM. Microenvironmental regulation of cancer stem cell phenotypes. Curr Stem Cell Res Ther (2012) 7(3):197–216. 10.2174/157488812799859838 22329582

[90] Axelson H Fredlund E Ovenberger M Landberg G Påhlman S Hypoxia-induced dedifferentiation of tumor cells – a mechanism behind heterogeneity and aggressiveness of solid tumors. Semin Cell Dev Biol (2005) 16(4–5):554–63. 10.1016/j.semcdb.2005.03.007 16144692

[91] Platet N Liu SY Atifi ME Oliver L Vallette FM Berger F Influence of oxygen tension on CD133 phenotype in human glioma cell cultures. Cancer Lett (2007) 258(2):286–90. 10.1016/j.canlet.2007.09.012 17977646

[92] Keith B Simon MC. Hypoxia-inducible factors, stem cells, and cancer. Cell (2007) 129(3):465–72. 10.1016/j.cell.2007.04.019 17482542 PMC3150586

[93] Peng F Wang JH Fan WJ Meng YT Li MM Li TT Glycolysis gatekeeper PDK1 reprograms breast cancer stem cells under hypoxia. Oncogene (2018) 37(8):1119. 10.1038/onc.2017.407 29251717 PMC5851083

[94] Lee SY Jeong EK Ju MK Jeon HM Kim MY Kim CH Induction of metastasis, cancer stem cell phenotype, and oncogenic metabolism in cancer cells by ionizing radiation. Mol Cancer (2017) 16(1):10. 10.1186/s12943-016-0577-4 28137309 PMC5282724

[95] Bhowmik SK Ramirez-Peña E Arnold JM Putluri V Sphyris N Michailidis G EMT-induced metabolite signature identifies poor clinical outcome. Oncotarget (2015) 6(40):42651–60. 10.18632/oncotarget.4765 26315396 PMC4767460

[96] Bonuccelli G Tsirigos A Whitaker-Menezes D Pavlides S Pestell RG Chiavarina B Ketones and lactate “fuel” tumor growth and metastasis: evidence that epithelial cancer cells use oxidative mitochondrial metabolism. Cell Cycle (2010) 9(17):3506–14. 10.4161/cc.9.17.12731 20818174 PMC3047616

[97] LaBarge MA. The difficulty of targeting cancer stem cell niches. Clin Cancer Res (2010) 16(12):3121–9. 10.1158/1078-0432.CCR-09-2933 20530700 PMC3182451

[98] Moore KA Lemischka IR. Stem cells and their niches. Science (2006) 311(5769):1880–5. 10.1126/science.1110542 16574858

[99] Cuyas E Corominas-Faja B Menendez JA. The nutritional phenome of EMT-induced cancer stem-like cells. Oncotarget (2014) 5(12):3970–82. 10.18632/oncotarget.2147 24994116 PMC4147299

[100] Aguilar E Marin de Mas I Zodda E Marin S Morrish F Selivanov V Metabolic reprogramming and dependencies associated with epithelial cancer stem cells independent of the epithelial-mesenchymal transition program. Stem Cells (2016) 34(5):1163–76. 10.1002/stem.2286 27146024 PMC4860823

[101] Chen EI Hewel J Krueger JS Tiraby C Weber MR Kralli A Adaptation of energy metabolism in breast cancer brain metastases. Cancer Res (2007) 67(4):1472–86. 10.1158/0008-5472.CAN-06-3137 17308085

[102] Sosa MS Bragado P Aguirre-Ghiso JA. Mechanisms of disseminated cancer cell dormancy: an awakening field. Nat Rev Cancer (2014) 14(9):611–22. 10.1038/nrc3793 25118602 PMC4230700

[103] Davidson SM Papagiannakopoulos T Olenchock BA Heyman JE Keibler MA Luengo A Environment impacts the metabolic dependencies of ras-driven non-small cell lung cancer. Cell Metab (2016) 23(3):517–28. 10.1016/j.cmet.2016.01.007 26853747 PMC4785096

[104] Abraham BK Fritz P McClellan M Hauptvogel P Athelogou M Brauch H. Prevalence of CD44+/CD24-/low cells in breast cancer may not be associated with clinical outcome but may favor distant metastasis. Clin Cancer Res (2005) 11(3):1154–9. 15709183

[105] Glinsky GV Berezovska O Glinskii AB. Microarray analysis identifies a death-from-cancer signature predicting therapy failure in patients with multiple types of cancer. J Clin Invest (2005) 115(6):1503–21. 10.1172/JCI23412 15931389 PMC1136989

[106] Moitra K Lou H Dean M. Multidrug efflux pumps and cancer stem cells: insights into multidrug resistance and therapeutic development. Clin Pharmacol Ther (2011) 89(4):491–502. 10.1038/clpt.2011.14 21368752

[107] Serafino A Zonfrillo M Andreola F Psaila R Mercuri L Moroni N CD44-targeting for antitumor drug delivery: a new SN-38-hyaluronan bioconjugate for locoregional treatment of peritoneal carcinomatosis. Curr Cancer Drug Targets (2011) 11(5):572–85. 10.2174/156800911795655976 21486216

[108] Mattheolabakis G Milane L Singh A Amiji MM. Hyaluronic acid targeting of CD44 for cancer therapy: from receptor biology to nanomedicine. J Drug Target (2015) 23(7–8):605–18. 10.3109/1061186X.2015.1052072 26453158

[109] Deonarain MP Kousparou CA Epenetos AA. Antibodies targeting cancer stem cells: a new paradigm in immunotherapy? MAbs (2009) 1(1):12–25. 10.4161/mabs.1.1.7347 20046569 PMC2715180

[110] Tamada M Nagano O Tateyama S Ohmura M Yae T Ishimoto T Modulation of glucose metabolism by CD44 contributes to antioxidant status and drug resistance in cancer cells. Cancer Res (2012) 72(6):1438–48. 10.1158/0008-5472.CAN-11-3024 22293754

[111] Annibaldi A Widmann C. Glucose metabolism in cancer cells. Curr Opin Clin Nutr Metab Care (2010) 13(4):466–70. 10.1097/MCO.0b013e32833a5577 20473153

[112] Krasnov GS Dmitriev AA Snezhkina AV Kudryavtseva AV. Deregulation of glycolysis in cancer: glyceraldehyde-3-phosphate dehydrogenase as a therapeutic target. Expert Opin Ther Targets (2013) 17(6):681–93. 10.1517/14728222.2013.775253 23445303

[113] Ceradini DJ Kulkarni AR Callaghan MJ Tepper OM Bastidas N Kleinman ME Progenitor cell trafficking is regulated by hypoxic gradients through HIF-1 induction of SDF-1. Nat Med (2004) 10(8):858–64. 10.1038/nm1075 15235597

[114] Roesch A Vultur A Bogeski I Wang H Zimmermann KM Speicher D Overcoming intrinsic multidrug resistance in melanoma by blocking the mitochondrial respiratory chain of slow-cycling JARID1B(high) cells. Cancer Cell (2013) 23(6):811–25. 10.1016/j.ccr.2013.05.003 23764003 PMC3810180

[115] Lamb R Ozsvari B Lisanti CL Tanowitz HB Howell A Martinez-Outschoorn UE Antibiotics that target mitochondria effectively eradicate cancer stem cells, across multiple tumor types: treating cancer like an infectious disease. Oncotarget (2015) 6(7):4569–84. 10.18632/oncotarget.3174 25625193 PMC4467100

[116] Lamb R Harrison H Hulit J Smith DL Lisanti MP Sotgia F. Mitochondria as new therapeutic targets for eradicating cancer stem cells: quantitative proteomics and functional validation via MCT1/2 inhibition. Oncotarget (2014) 5(22):11029–37. 10.18632/oncotarget.2789 25415228 PMC4294326

[117] An H Kim JY Oh E Lee N Cho Y Seo JH. Salinomycin promotes anoikis and decreases the CD44+/CD24- stem-like population via inhibition of STAT3 activation in MDA-MB-231 cells. PLoS One (2015) 10(11):e0141919. 10.1371/journal.pone.0141919 26528725 PMC4631341

[118] Chu DJ Yao DE Zhuang YF Hong Y Zhu XC Fang ZR Azithromycin enhances the favorable results of paclitaxel and cisplatin in patients with advanced non-small cell lung cancer. Genet Mol Res (2014) 13(2):2796–805. 10.4238/2014.April.14.8 24782093

[119] Mayer MJ Klotz LH Venkateswaran V. Metformin and prostate cancer stem cells: a novel therapeutic target. Prostate Cancer Prostatic Dis (2015) 18(4):303–9. 10.1038/pcan.2015.35 26215782

[120] Jung JW Park SB Lee SJ Seo MS Trosko JE Kang KS. Metformin represses self-renewal of the human breast carcinoma stem cells via inhibition of estrogen receptor-mediated OCT4 expression. PLoS One (2011) 6(11):e28068. 10.1371/journal.pone.0028068 22132214 PMC3223228

[121] Vazquez-Martin A Oliveras-Ferraros C Cufí S Del Barco S Martin-Castillo B Menendez JA. Metformin regulates breast cancer stem cell ontogeny by transcriptional regulation of the epithelial-mesenchymal transition (EMT) status. Cell Cycle (2010) 9(18):3807–14. 10.4161/cc.9.18.13131 20890129

[122] Mullen AR Wheaton WW Jin ES Chen PH Sullivan LB Cheng T Reductive carboxylation supports growth in tumour cells with defective mitochondria. Nature (2011) 481(7381):385–8. 10.1038/nature10642 22101431 PMC3262117

[123] Subramaniam D Nicholes ND Dhar A Umar S Awasthi V Welch DR 3,5-bis(2,4-difluorobenzylidene)-4-piperidone, a novel compound that affects pancreatic cancer growth and angiogenesis. Mol Cancer Ther (2011) 10(11):2146–56. 10.1158/1535-7163.MCT-11-0399 21890747 PMC3213278

[124] Dupuy F Tabariès S Andrzejewski S Dong Z Blagih J Annis MG PDK1-dependent metabolic reprogramming dictates metastatic potential in breast cancer. Cell Metab (2015) 22(4):577–89. 10.1016/j.cmet.2015.08.007 26365179

[125] Cheong JH Park ES Liang J Dennison JB Tsavachidou D Nguyen-Charles C Dual inhibition of tumor energy pathway by 2-deoxyglucose and metformin is effective against a broad spectrum of preclinical cancer models. Mol Cancer Ther (2011) 10(12):2350–62. 10.1158/1535-7163.MCT-11-0497 21992792 PMC3237863

[126] Issaq SH Teicher BA Monks A. Bioenergetic properties of human sarcoma cells help define sensitivity to metabolic inhibitors. Cell Cycle (2014) 13(7):1152–61. 10.4161/cc.28010 24553119 PMC4013165

[127] Korkaya H Liu S Wicha MS. Breast cancer stem cells, cytokine networks, and the tumor microenvironment. J Clin Invest (2011) 121(10):3804–9. 10.1172/JCI57099 21965337 PMC3223613

[128] Garvalov BK Acker T. Cancer stem cells: a new framework for the design of tumor therapies. J Mol Med (Berl) (2011) 89(2):95–107. 10.1007/s00109-010-0685-3 20890588

[129] Gu W Yeo E McMillan N Yu C. Silencing oncogene expression in cervical cancer stem-like cells inhibits their cell growth and self-renewal ability. Cancer Gene Ther (2011) 18(12):897–905. 10.1038/cgt.2011.58 21904396

[130] Moserle L Ghisi M Amadori A Indraccolo S. Side population and cancer stem cells: therapeutic implications. Cancer Lett (2010) 288(1):1–9. 10.1016/j.canlet.2009.05.020 19523754

[131] Brandt WD Matsui W Rosenberg JE He X Ling S Schaeffer EM Urothelial carcinoma: stem cells on the edge. Cancer Metastasis Rev (2009) 28(3–4):291–304. 10.1007/s10555-009-9187-6 20012172 PMC2930269

